# Supplementary material for: Safety and efficacy of esaxerenone in Japanese hypertensive patients with heart failure with reduced ejection fraction: A retrospective study
Source: PLoS One. 2021 Nov 8;16(11):e0259485. doi: 10.1371/journal.pone.0259485 (PMC8575262; doi:10.1371/journal.pone.0259485)
Supplement: Attachment — Submitted filename: Response to Reviewers.docx [file pone.0259485.s002.docx]

**Response to Reviewers**

**Reviewers' comments:**

**Reviewer's Responses to Questions**

**Comments to the Author**

**1. Is the manuscript technically sound, and do the data support the conclusions?**

**The manuscript must describe a technically sound piece of scientific research with data that supports the conclusions. Experiments must have been conducted rigorously, with appropriate controls, replication, and sample sizes. The conclusions must be drawn appropriately based on the data presented.**

**Reviewer #1: Partly**

**Reviewer #2: Yes**

> We thank the reviewers for their responses.

**2. Has the statistical analysis been performed appropriately and rigorously?**

**Reviewer #1: Yes**

**Reviewer #2: Yes**

> We thank the reviewers for their responses.

**3. Have the authors made all data underlying the findings in their manuscript fully available?**

**The PLOS Data policy requires authors to make all data underlying the findings described in their manuscript fully available without restriction, with rare exception (please refer to the Data Availability Statement in the manuscript PDF file). The data should be provided as part of the manuscript or its supporting information, or deposited to a public repository. For example, in addition to summary statistics, the data points behind means, medians and variance measures should be available. If there are restrictions on publicly sharing data—e.g. participant privacy or use of data from a third party—those must be specified.**

**Reviewer #1: Yes**

**Reviewer #2: Yes**

> We thank the reviewers for their responses.

**4. Is the manuscript presented in an intelligible fashion and written in standard English?**

**PLOS ONE does not copyedit accepted manuscripts, so the language in submitted articles must be clear, correct, and unambiguous. Any typographical or grammatical errors should be corrected at revision, so please note any specific errors here.**

**Reviewer #1: Yes**

**Reviewer #2: Yes**

> We thank the reviewers for their responses.

**5. Review Comments to the Author**

**Please use the space provided to explain your answers to the questions above. You may also include additional comments for the author, including concerns about dual publication, research ethics, or publication ethics. (Please upload your review as an attachment if it exceeds 20,000 characters)**

**Reviewer #1: Comment: Iwahana et al. conducted a retrospective study to investigate the safety and the efficacy of esaxerenone, a novel non-steroidal MRB in subjects with hypertension and heart failure with reduced ejection fraction. After a short term (35 days) and mid term (167 days) follow-up, no serious adverse event (hypotension, hyperkalemia, hospitalization for health failure) was observed. A small number (n=7) of patients showed a worsening of renal function, though superimposable with the one reported in other major trials and of mild clinical relevance. A slight, non-significant, elevation of potassium was also detected. In terms of efficacy, however, a consistent over time BNP reduction was assessed.**

**Albeit with the limitations implicit in a small retrospective study, the novelty of the drug under observation and the significance of the clinical context make the findings and the paper relevant. However, the authors should amend some flaws, mainly in the discussion section, to make it suitable for publication.**

**Major**

**Limitations: the main limits of this work lie in the combination of being a retrospective study with a very small sample size and no control group. Indeed, this substantially hinder the appreciation of an efficacy effect, and this should be wider discussed in the “study limitation”. Also, cardiovascular events were defined as outcome. However it seems, from the text, that no event was reported (except that one in Figure 1), but this is probably due to the short follow-up. Taken this into account, the authors should also try to considerably mitigate their statement regarding efficacy. Furthermore, the small and specific (Japanese) population limits the generalization of the results. This should be better discussed and, probably, also added in the title. In general, I feel that, as original research article, the limitations of the work are various and of major relevance and should be better addressed.**

> We thank the reviewer for this insightful comment. As the reviewer indicated, this retrospective observational study has several limitations; the limited population and the shortness of the observation period. Indeed, esaxerenone is currently available only in Japan. Moreover, we were unable to follow-up the patients long-term, which seems to be one reason why the number of AEs was small. In previous studies regarding MRBs, however, K^+^ elevation and eGFR decrease were reported to be most conspicuous within the first month. We intended to investigate mainly the safety of the drug; thus, our results have a certain significance regarding safety. A large-scale, multinational, and randomized control study is required to resolve the potential bias. We added several sentences and a reference [23] in the **Study limitations** (Line 256-262).

Also, we revised the title of the manuscript to “Safety and Efficacy of Esaxerenone in Japanese Hypertensive Patients with Heart Failure with Reduced Ejection Fraction: a retrospective study”.

**Minor**

**Line 180: modify "initial assessment" with "short term assessment".**

> We thank the reviewer for this valuable comment. We modified the sentence (Line 185), accordingly.

**Lines 229-231: To provide a stronger meaning of safety, I suggest modifying the sentence in “****In the present study, no patient presented with hypotension, even if in some patients SBP had been already normalized at baseline with other antihypertensive agents.”**

> We thank the reviewer for this valuable comment. Given that we agree with your suggestion, we revised the sentence (Lines 242-244).

**Lines 233-235: The paragraph is too short. As stated above, the authors should make at least a major effort here if they intend to comment about safety. Maybe some literature quantitative contextualization would be helpful here (i.e. comparison of the effect on BNP levels - absolute or percentage reduction- with other MRB on similar cohorts). I suggest also implementing the data on hospitalization for heart failure here, and also discussing the short follow-up time.**

> We thank the reviewer for this pertinent comment. We referred to two appropriate articles. According to a review analyzing 16 phase III chronic HF trials, therapy-related changes in natriuretic peptides appeared modestly correlated with longer-term therapeutic effects on hospitalization for HF, but not with effects on all-cause mortality. Regarding MRBs, the ARTS-HF study reported that 30-40% of HF patients with diabetes mellitus and/or chronic kidney disease treated with eplerenone or finerenone presented with a >30% reduction of NT-proBNP. Esaxerenone in the present study demonstrated comparable BNP reduction to other MRBs, which suggests that esaxerenone is potentially beneficial for HFrEF. We added this content in Lines 247-253.

**Reviewer #2: The Iwahana and co-authors manuscript aimed to investigate the short- to mid-term safety and efficacy of esaxerenone in hypertensive patients with heart failure with reduced ejection fraction (HFrEF). The present study was a single-center, retrospective observational study conducted in Chiba University Hospital, included a small number of patients and part of then were converted to esaxerenone from another MRB – this observation was pointed in the discussion section. Although these points, the present study suggested that esaxerenone is safe for hypertensive patients with HFrEF, with a low incidence of hyperkalemia and hypotension. In addition, esaxerenone was found to be beneficial for heart failure in terms of BNP level reduction.**

**Major comments:**

**1. To reinforce the esaxerenone efficacy for heart failure the authors could compare the echocardiographic data before and after short- to mid-term esaxerenone treatment. Please, analyze these echocardiographic parameters.**

> We thank the reviewer for this important suggestion. A total of 40 patients underwent an echocardiography 160±82 days after starting esaxerenone. Comparison between pre- and post- echocardiographic parameters presented with LV reverse remodeling (LVDD: 61.8±7.4 mm to 58.9±8.7 mm, p=0.002, LVDS: 52.7±8.1 mm to 48.1±10.7 mm, p<0.001, LVEF 30.8±6.0% to 38.5±10.3%, p<0.001) and left atrial volume index reduction (LAVI: 46.5±21.1 ml/m^2^ to 39.1±17.0 ml/m^2^, p=0.02). E/A and E/e’ tended to improve, but not significantly (E/A 1.19±1.00 to 0.88±0.30, p=0.12, E/e’ 13.0±7.0 to 10.8±4.2, p=0.11). These results suggest that esaxerenone is potentially beneficial for systolic and, perhaps, diastolic function. However, we cannot conclude that these beneficial changes of echocardiography were entirely due to the efficacy of esaxerenone, due to the study design; retrospective, observational, and non-randomized, without a control group. In some patients, other cardioprotective agents were added at close timing. Therefore, we did not present with the comparison of pre- and post- echocardiographic data. A randomized and placebo control study is required to resolve this issue.

**2. The authors could include diastolic parameters in the echocardiographic analysis and evaluate the role of esaxerenone in these parameters.**

> We thank the reviewer for this valuable comment. We added the baseline echocardiographic data including LAVI, E/A, and E/e’ (Table 1).

**Minor comments:**

**1. Please, add each Statistical analysis applied in the table and figure legends.**

**2. Please, change the supplemental figure to the regular figure 4.**

> We thank the reviewer for this valuable comment. We revised the table and figure legends, and changed the supplemental figure to ‘Fig 4’.

**6. PLOS authors have the option to publish the peer review history of their article (what does this mean?). If published, this will include your full peer review and any attached files.**

**If you choose “no”, your identity will remain anonymous but your review may still be made public.**

**Do you want your identity to be public for this peer review? For information about this choice, including consent withdrawal, please see our Privacy Policy.**

**Reviewer #1: No**

**Reviewer #2: No**

Thank you once again for your valuable comments and suggestions. We are hopeful that our revisions help to improve your opinion of our work.
